# Foliar Application of Zinc Oxide Nanoparticles Promotes Drought Stress Tolerance in Eggplant (*Solanum melongena* L.)

**DOI:** 10.3390/plants10020421

**Published:** 2021-02-23

**Authors:** Wael M. Semida, Abdelsattar Abdelkhalik, Gamal. F. Mohamed, Taia A. Abd El-Mageed, Shimaa A. Abd El-Mageed, Mostafa M. Rady, Esmat F. Ali

**Affiliations:** 1 Horticulture Department, Faculty of Agriculture, Fayoum University, Fayoum 63514, Egypt; wms00@fayoum.edu.eg (W.M.S.); aga04@fayoum.edu.eg (A.A.); 2 Botany Department, Faculty of Agriculture, Fayoum University, Fayoum 63514, Egypt; gfm00@fayoum.edu.eg; 3 Soil and Water Department, Faculty of Agriculture, Fayoum University, Fayoum 63514, Egypt; taa00@fayoum.edu.eg; 4 Agronomy Department, Faculty of Agriculture, Fayoum University, Fayoum 63514, Egypt; sa1944@fayoum.edu.eg; 5 Department of Biology, College of Science, Taif University, P.O. Box 11099, Taif 21944, Saudi Arabia; a.esmat@tu.edu.sa

**Keywords:** zinc oxide nanoparticles, deficit irrigation, water productivity, photosynthetic efficiency, growth and productivity

## Abstract

Water shortage and salinity are major challenges for sustaining global food security. Using nutrients in the nano-scale formulation including zinc oxide nanoparticles (ZnO NP) is a novel fertilization strategy for crops. In this study, two field-based trials were conducted during 2018 and 2019 to examine the influence of three ZnO NP concentrations (0, 50, and 100 ppm) in eggplant grown under full irrigation (100 of crop evapotranspiration; ETc) and drought stress (60% of ETc). Plant growth, yield, water productivity (WP), physiology, biochemistry, and anatomy responses were evaluated. Drought stress significantly decreased membrane stability index (MSI), relative water content (RWC), and photosynthetic efficiency, thus hampered eggplant growth and yield. In contrast, exogenous ZnO NP to water-stressed eggplant resulted in increased RWC and MSI associated with improved stem and leaf anatomical structures and enhanced photosynthetic efficiency. Under drought stress, supplementation of 50 and 100 ppm ZnO NP improved growth characteristics and increased fruit yield by 12.2% and 22.6%, respectively, compared with fully irrigated plants and nonapplied ZnO NP. The highest water productivity (WP) was obtained when eggplant was irrigated with 60% ETc and foliarly treated with 50 or 100 ppm of ZnO NP, which led to 50.8–66.1% increases in WP when compared with nontreated fully irrigated plants. Collectively, these findings demonstrated that foliar spraying ZnO NP gives the utility for alleviating drought stress effects on eggplant cultivated in saline soil.

## 1. Introduction

Sustainable agricultural development depends on the type and supply of irrigation water worldwide, given that agriculture consumes approximately 69% of the total freshwater [1]. Universally, water and food production is closely related, with nearly 40% of agricultural production around the world coming from irrigated cropland [2]. In the Mediterranean region, water resources are scarce, which calls for the need to reassess current water use practices. The increasing population growth leads to an increase in the demand for food, which increases the irrigated area, which has increased eight-fold over the past century [3,4,5]. These restrictions and risks to food security will be massive, particularly with the expected climate change, causing increased competition for water resources between different sectors [6,7]. To weaken global changes in the future and ensure food security, many attempts are being made through investigations to boost water productivity (WP). Therefore, effective innovations in irrigation and management techniques are needed to achieve more efficient and logical use of water [8,9,10,11,12]. In this concern, deficit irrigation (DI) is used as a practice to save water by adding less water than the irrigation water requirement [3,13]. 

Eggplant (*Solanum melongena* L.) is a distinct crop worldwide, with a cultivated area of 1.86 million ha, producing approximately 54 million Mg. Globally, Egypt ranked third of the largest eggplant producers accounting approximately 2.6% of the world’s production [14]. Eggplant required uniform soil moisture for obtaining important marketable yield [15,16]. Although eggplant was considered to be moderately tolerant to water stress [17], it is difficult to apply water deficits, especially in arid and semiarid areas with saline soil without yield reduction. Withholding irrigation to four varieties of eggplant strongly reduced the fresh weight of different plant parts (roots, stem, and leaves) and leaf water content as well as decreased chlorophyll content [18]. Eggplant fruit yield decreased up to 60% when water deficits increased from 20% to 40% of the field capacity [19]. Deficiencies in nutrients absorption and transport due to water stress, resulted in loss of crop yield. Further, under alkaline soil conditions, the absorption and transfer of nutrients from the roots to the leaves decrease, especially microelements [20].

Zinc (Zn) is a micro-nutrient having important roles in the growth of all crop plants. Zn is involved in the activity of various enzymes (RNA and DNA polymerases, dehydrogenases, transphosphorylases, and proteinase), as well as contributes to the maintenance of the membrane structure and cell division, chlorophyll biosynthesis, and improves plant photosynthetic apparatus [20,21,22]. Foliar-applied microelements are more convenient for plant response at the field scale, as it is environmentally friendly compared to soil application which may show toxicity upon adding the same microelements [21,23]. Micronutrients have been shown to alleviate water stress in plants by enhancing WP, maintaining cell integrity, and detoxifying drought-induced free radicals [24,25].

In recent years, the incorporation of nanomaterials products in many sectors including nanofertilizers is gaining interest. Nanoparticles (NP) have novel properties as a result of their small size (below 100 nm at least in one dimension) which results in high surface areas and surface charges, therefore, NP are more reactive than their bulk scale counterparts [25,26]. Nanofertilizers are used to gradually release nutrients while minimizing soil pollution [27]. These nanoscale fertilizers are an approach that makes nutrients available to plant leaves, thus increasing the efficiency of plant nutrient uptake [28]. ZnO NP have been reported to alleviate oxidative damage in various crops [29,30,31]. ZnO NP reduced malondialdehyde (MDA) level and enhanced CAT and SOD activities in stressed *Leucaena leucocephala* [30]. Similar results were also reported by in green pea and sugar beet [31]. One of the benefits of nanoscale fertilizers is to minimize the addition rate of nutrients, thus saving the input costs and sustainably minimizing the environmental footprint of chemical fertilizers [32]. Due to their small size, nanofertilizers have a higher and faster translocation between plant parts, which increases nutrient efficiency [33].

Conventional Zn fertilizer in the form of ZnSO_4_·7H_2_O has a very low Zn use efficiency (1–5%). However, zinc oxide nanoparticles (ZnO NP) are systematically evaluated in plants to enhance their ability to modulate crop productivity and nutrient use efficiency [32]. It has been previously demonstrated that using nanofertilizers has the potential to promote drought stress tolerance in several crops; soybean [20], maize [34], and wheat [32]. According to [22], spraying ZnO NP only or in combination with Cu NP and Mn NP increased basil plant growth, chlorophyll content, as well as enhanced antioxidant activity. ZnO NP amended drought-stressed sorghum plant, increased green yield up to 183%, and improved total N and K acquisition by the plant [25].

A combination of deficit irrigation and the application of nanofertilizers may have the potential to provide significant water-savings and improve the WP in eggplant. At the field scale, eggplant responses to combined deficit irrigation and foliar application of ZnO NP grown under salt-alkaline soil are not yet fully investigated. Therefore, this research aimed to explore the potential effect of foliar-applied ZnO NP to ameliorate the drought stress on eggplant. Further, studying their impact on eggplant growth, yield, WP, tissue water status, photosynthetic efficiency, nutrients contents, and anatomical responses.

## 2. Materials and Methods

### 2.1. Experimental Site 

Two trials were consecutively conducted during the summer season of 2018 and 2019 (5 April to 29 August) at El Fayoum region (latitudes 29°02′ and 29°35′ N and longitudes 30°23′ and 31°05′ E), Egypt. Average climatic data of this region during the two growing seasons are given in Table 1. A typic torripsamment, siliceous, hyperthermic, loamy sand [35], and its physicochemical characteristics were evaluated [36,37] and displayed in Table 2 and Table 3.

### 2.2. Experimental Design and Treatment Applications

**Figure 1:**
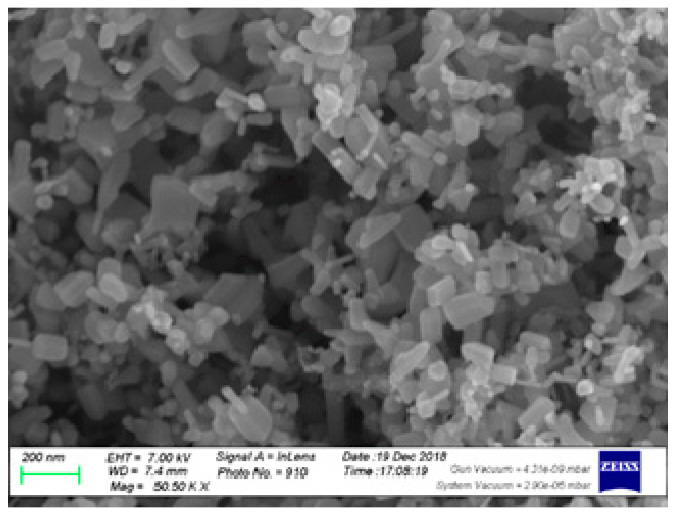
TEM image of ZnO nanoparticles (ZnO NP).

The arrangement of the trial was a split-plot system in a randomized complete block design (RCBD) with three replicates. Twice irrigation regimes (full irrigation, FI (100% of crop evapotranspiration; ETc) and deficit irrigation, DI (60% of ETc)) were applied to the main plots and the subplots with three ZnO NP concentrations (0, 50, and 100 ppm) that were foliarly applied at two times; 30 days after transplanting and 2 weeks later. Thus, six treatments were used as follows: I_100_ (100% of ETc), I_60_ (60% of ETc), I_100_ + ZnO NP_50_ (50 ppm nanoscale ZnO), I_100_ + ZnO NP_100_ (I_100_ + 100 ppm nanoscale ZnO), I_60_ + ZnO NP_50_, and I_60_ + ZnO NP_100_. Figure 1 shows the TEM image of ZnO nanoparticles (ZnO NP).

The experimental area was divided into plots. The area of each plot was 15 m long × 0.70 m row width (10.5 m^2^). Plants were spaced about 30 cm between every two plants in rows, each row containing 50 plants. Eggplant transplants (cv. hybrid Soma^®^) of 30 days old, secured from nurseries of the Ministry of Agriculture, were transplanted at a rate of one per emitter. The drip irrigation system was assigned at one line and one dripper for each plant, giving 3.6 L of saline irrigation water (1.88 dS m^−1^; Table 4) per hour. Seedlings were transplanted on 5 April and the trial was ended on 29 August, (in both seasons). Nonirrigated areas 3 m wide were designated as separation boundaries between each two irrigation treatments. Seven days after the transplanting, irrigation treatments were started. The agronomic practices for commercial eggplant crop production, including pest, weed, and disease control were followed as recommended.

### 2.3. Irrigation Water Applied (IWA)

Eggplant seedlings were watered with different quantities of irrigation water at intervals of 2 days till the end of trial. The water requirements of the crop (ETc) were given as determined using class A pan equation [38]:

ET_c_ = E_pan_ × K_pan_ × K_c_

where ETc is the crop water requirement (mm day^−1^), E_pan_ is the evaporation from the Class A pan (mm day^−1^), K_pan_ the Pan coefficient [38], and K_c_ is the crop coefficient. 

Irrigation water application (IWA) was determined by using the following formula:

IWA = (A × ETc × Ii × Kr) ÷ [Ea × 1000 × (1 − LR)]

where IWA = irrigation water applied in m^3^, Ea = application efficiency%, LR = leaching requirements, A = plot area in m^2^, ETc = crop water requirements in mm per day, Ii = irrigation intervals (day), and Kr = covering factor.

### 2.4. Measurements

At the end of the trial, 10 plants were taken, randomly, from every experimental plot and assessed for growth characteristics. The fourth fully expanded leaves from the apex of stem (and its internodes) which emerged after drought imposition were taken per plant for assessing the morphological, physiological, macro and micronutrients and anatomical parameters.

#### 2.4.1. Morphological Parameters and Yield and its Components

Plant height, stem diameter, and shoot fresh and dry weight were recorded (cm) at the end of the trial. Plant leaf area (cm^2^) was measured using the relationship of leaf area–leaf weight as demonstrated by [36] withsome modifications. Leaf surface was thoroughly washed in running tap water followed by washing with double-distilled water, thereafter 10–20 leaf disks (10–20 cm^2^) were dried in an oven at 85 °C for 24 h to get disks dry weight (DDW). Total leaf area plant^−1^ was calculated using the following formula:

Total leaf area plant^−1^ = (LDW ÷ DDW) × DA (1)

where LDW is the total leaf dry weight (g), DDW is the disks dry weight (g), and DA is the discs area. 

A total of 50 days after transplanting, five plants were specified from each experimental plot to weekly record the average of fruit length (cm), number of fruits plant^−1^, fruit weight (g), and total yield (t ha^−1^). 

#### 2.4.2. Physiological Measurements

Relative water content percentage (RWC, %) was determined based on fresh (FM in g), turgid (TM in g), and dry weights (DM in g) of leaf discs. After measuring FM of fresh leaves discs, they were placed into containers (slightly longer than the sample) with distilled for 24 h until a constant weight (the adhering water of the leaves was botted with absorbent paper toweling). TM was measured for each sample. DM was obtained after drying these leaves at 70 °C in an oven for 72 h to a constant weight. Relative water content percentage (RWC, %) was determined following the [39] equation:

RWC (%) = [(FM − DM) ÷ (TM − DM)] × 100

Based on the electrical conductivity of two leaf samples devoid of midribs, heated at two different temperatures, 40 and 100 °C for 30 and 10 min (C1 and C2), respectively, the percentage of membrane stability index (MSI, %) was determined following the [40] equation:

MSI (%) = [1 − (C1 ÷ C2)] × 100

A portable fluorometer (Handy PEA, Hansatech Ltd., Kings Lynn, UK) was used to assess chlorophyll ‘*a*’ fluorescence. The equation *F_v_*/*F_m_* = (*F_m_* − *F*_0_) ÷ *F_m_* [41] was practiced to calculate the PSII maximum quantum yield. Based on the equal absorption, the equation included in [42] was also practiced to calculate the index of photosynthetic performance (PIABS). SPAD meter (SPAD-502-2900) was used to measure relative chlorophyll index of the eggplant. Possessing a 0.98 emissivity and a spectral response range of 8–14 μm, an infrared thermometer (Fluk 574, Everett, WA, USA) was functioned to perform the measurements of the canopy temperature. 

Water productivity (WP) was calculated as mentioned in [43]:

WP = [fruit yield (kg ha^−1^)] ÷ [water applied (m^3^ ha^−1^)]

### 2.5. Macro- and Micronutrients Assessments

The assessments of plant tissue contents of nutrients, N, P, K, Fe, Mn, and Zn were assessed in dry, fine ground eggplant leaves (*n* = 10). A micro-Kjeldahl (Medic. Instr. Co., Ningbo, China) apparatus was functioned to determine N content following the methods in [44]. Based on the method in [45], P content was assessed with molybdenum blue, H_2_MoO_7_S, 8% (*w*/*v*) NaHSO_3_-H_2_SO_4_, and diluted H_2_MoO_7_S as standard reagents. An Atomic Absorption Spectrophotometer (Perkin-Elmer, Model 3300) was used to assess the contents of Zn, Mn, and Fe in plant leaves as described in [46]. 

### 2.6. Anatomical Features

Leaf and stem specimens were secured from the middle internode with its leaf blade. The selected specimens were chosen from plants at the flowering stage for killing and fixing for 48 h in 100 mL of F.A.A. solution containing 50 mL of C_2_H_5_OH (95%), 5 mL of glacial acetic acid, 10 mL of formalin, in addition to 35 mL of distilled water. Then, samples were exposed to washing using C_2_H_5_OH (50%). Dehydration and clearance were then performed with normal butyl alcohol series, and embedded in paraffin wax (54–56 °C m.p). A rotary microtome was functioned to cut samples for 20 μm thick cross-sections that were adhesive (Haupt’s adhesive). The samples were then stained with the crystal violet–erythrosin combination [47]. Slide photography was performed and then read by micrometric eye lens to obtain various anatomical features expressed in µm.

### 2.7. Data Analysis

The GLM procedure of Gen Stat (version 11) (VSN International Ltd., Oxford, UK) was used to analyze the experimental data. The homogeneity test of error variance was conducted as stated in a method described by Gomez and Gomez [47]. Data from the two seasons were subjected to a combined analysis and, among the means, differences were compared by Duncan’s Multiple Range Test at 5% probability (*p* ≤ 0.05) level.

## 3. Results

### 3.1. Changes in Eggplant Growth by Foliar-Applied ZnO NP and Deficit Irrigation

Eggplant growth in terms of plant height, number of leaves per plant, stem diameter, fresh and dry weights of plant shoot, and total plant leaf area were significantly impacted by ZnO NP foliar application under deficit irrigation (DI) stress (Table 5). In this respect, DI reduced plant height by 16.3%, leaves number by 23.7%, stem diameter by 7.7%, shoot fresh weight by 25.8%, shoot dry weight 24.2%, and leaf area by 27.4% compared with fully irrigated plants. These growth traits significantly increased by foliar application of ZnO NP and these improvements were more pronounced under ZnO NP_(100)_. Exogenous application of ZnO NP alleviated the adverse impacts of DI stress on eggplant growth, in the sense that spraying with ZnO NP (50 or 100 ppm) to plants grown under DI gave similar or higher values than those growing under full irrigation without ZnO NP supply (FI + ZnO NP_(0)_).

### 3.2. Changes in Photosynthetic Efficiency and Tissue Water Status by Foliar-Applied ZnO NP and Deficit Irrigation

Performance index (PI) of the photosynthetic efficiency and relative chlorophyll index (SPAD) were decreased by 22.2% and 5.9%, respectively, under DI (60% of ETc) compared to plants grown under FI (Table 6). Comparing non-treated ZnO NP plants, the maximum quantum yield PSII of photochemistry (*F_v_*/*F_m_*), PI, and SPAD value were increased with ZnO NP (50 or 100 ppm). Application of ZnO NP (50 or 100 ppm) to drought-stressed plants increased *F_v_*/*F_m_*, PI, and SPAD value and recorded similar values to nontreated FI plants. Eggplant water status in terms of RWC and MSI were negatively influenced by water stress with 40%. However, both RWC and MSI were increased by 11.3% and 4.8% (on average), respectively, in ZnO NP-treated plants compared to those nontreated (Table 6). Spraying ZnO NP (50 or 100 ppm) alleviated the adverse effects of drought stress via increasing RWC and MSI as those observed under FI without ZnO NP application (Table 6).

### 3.3. Changes of Eggplant Yield and Water Productivity in Response to Foliar-Applied ZnO NP and Deficit Irrigation

Results of the average length of fruit, individual fruit weight, total number of fruits plant^−1^, total fruit yield, and WP in response to DI, exogenously applied ZnO NP, and their interaction are presented in Table 7. Reducing irrigation down to 60% ETc significantly decreased fruit length by 9.2%, fruit weight by 18.3%, and the number of fruits plant^−1^ by 8.0% relative to FI. ZnO NP-treated plants with 50 or 100 ppm ZnO NP showed the greatest length and weight of fruit, and total number of fruits plant^−1^ compared to nontreated plants. Spraying 100 ppm ZnO NP to drought-stressed eggplant showed similar values of fruit length and average fruit weight to eggplants subjected to FI without ZnO NP application (FI + ZnO NP_(0)_). However, water-stressed eggplant supplemented with 50 or 100 ppm ZnO NP produced higher fruit numbers by 25.6% and 33.1%, respectively, in comparison with FI + ZnO NP_(0)_.

**Figure 2:**
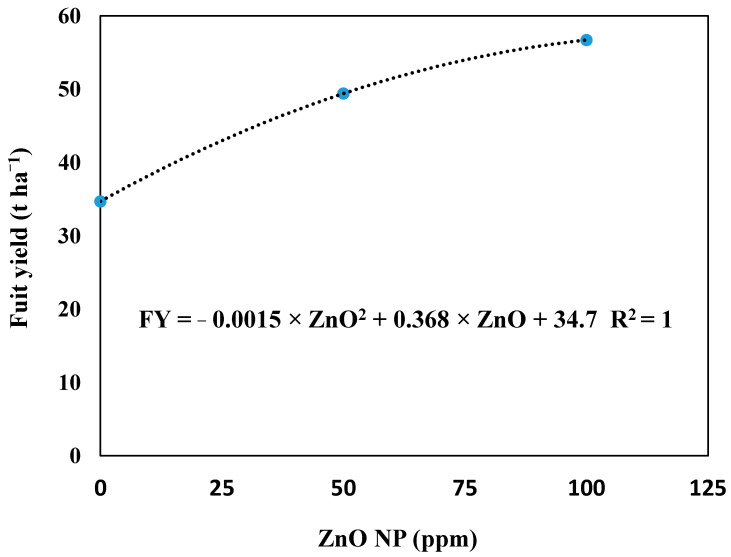
Regression analysis between concentrations of ZnO nanoparticles (ZnO NP) and fruit yield (t ha^−1^) of eggplant.

Water deficit exposure noticeably decreased fruit yield by 17% in comparison to FI, fruit yield was also affected by exogenous application of 50 or 100 ppm ZnO NP that increased by 42.4% and 63.4%, respectively, compared with DI × ZnO NP_(0)_. The greatest fruit yield corresponded with the integrative application of 100 ppm ZnO NP and FI treatment. However, combined exogenously applied 50 or 100 ppm of ZnO NP and water deficit at 60% ETc exhibited higher fruit yield by 12.2% and 22.6%, respectively, than those obtained with FI + ZnO NP_(0)_ treatment. As shown in Figure 2, the regression analysis among fruit yield and ZnO NP concentration was a curvilinear relationship, indicating increasing fruit yield with increasing ZnO NP concentration.

Results of Table 7 reported that the WP was significantly influenced by irrigation regimes, supplementation of ZnO NP, and their interaction. Under 40% water deficit, WP increased by 9.9% compared to FI. Whilst, exogenous ZnO NP application with 50 and 100 ppm increased WP by 49.1% and 69.8% compared to DI × ZnO NP_(0)_. The highest WP were obtained when plants were irrigated with 60% ETc and foliarly-sprayed with 50 or 100 ppm of ZnO NP, which led to 50.8–66.1% increases in WP when compared with FI + ZnO NP_(0)_.

### 3.4. Changes in Nutrients Contents in Response to Foliar-Applied ZnO NP and Deficit Irrigation

Macronutrients (i.e., N and K) and micronutrients (i.e., Zn, Mn, and Fe) of eggplant leaf tissues were significantly decreased under water shortage by 40%, whereas, P contents were not affected by the irrigation regimes (Table 8). Compared to nontreated ZnO NP, spraying ZnO NP (both 50 or 100 ppm) to eggplant increased their leaf tissue content of macronutrients like N, P, and K and micronutrients like Zn, Mn, and Fe (Table 8). In water-stressed eggplant leaf tissues, the macro and micronutrient concentrations were markedly increased by ZnO NP supplementation. Among ZnO NP application, DI × ZnO NP_(100)_ exhibited higher N by 21.6%, P by 91.4%, K by 9.4%, Zn by 65.0%, Mn by 27%, and Fe 6.6% when compared with FI × ZnO NP_(0)_ (Table 8).

### 3.5. Leaf and Stem Anatomical Responses to Foliar-Applied ZnO NP and Deficit Irrigation

**Figure 3:**
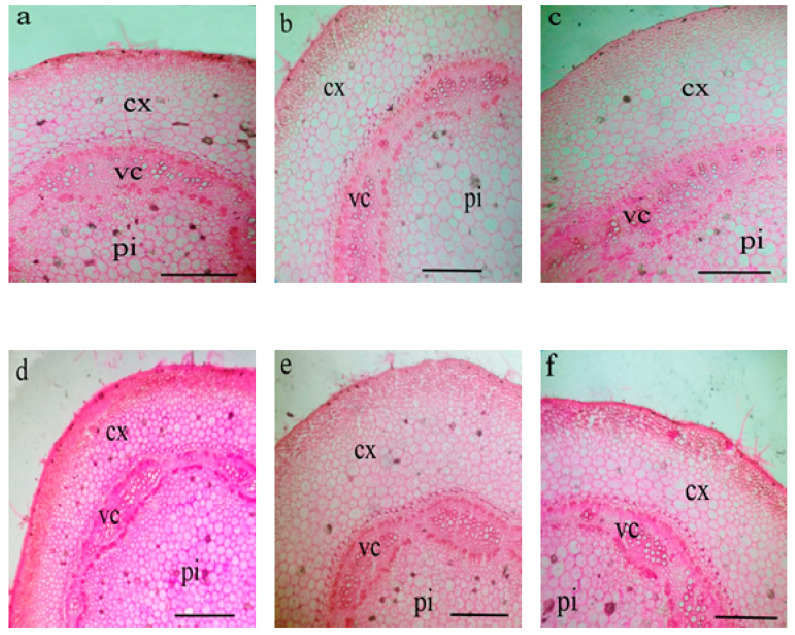
Transverse section in eggplant stem as influenced by foliar application with ZnO nanoparticles (ZnO NP) under full (FI) and deficit (DI) irrigation. (**a**) FI × ZnO NP_(0)_, (**b**) FI × ZnO NP_(50)_, (**c**) FI × ZnO NP_(100)_, (**d**) DI × ZnO NP_(0)_, (**e**) DI × ZnO NP_(50)_, (**f**) DI × ZnO NP_(100)_. Scale bar = 350 μm. (cx = cortex, vc = vascular cylinder, pi = pith).

**Figure 4:**
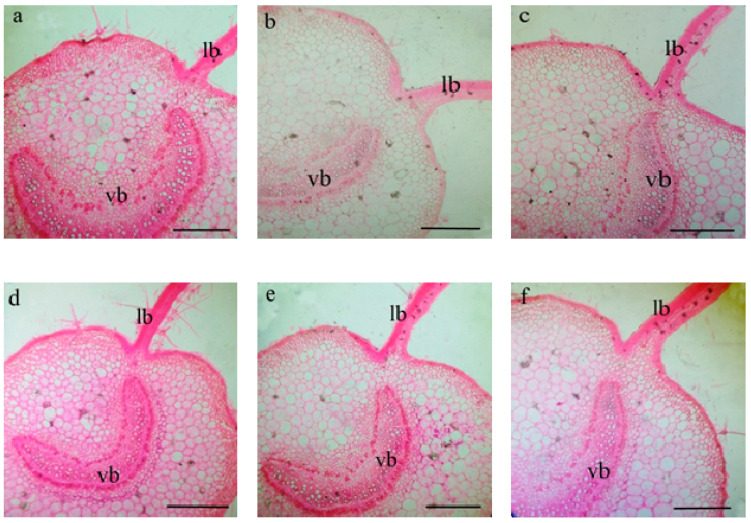
Transverse section in eggplant leaf as influenced by foliar application with ZnO nanoparticles (ZnO NP) under full (FI) and deficit (DI) irrigation. (**a**) FI × ZnO NP_(0)_, (**b**) FI × ZnO NP_(50)_, c: FI × ZnO NP_(100)_, (**d**) DI × ZnO NP_(0)_, (**e**) DI × ZnO NP_(50)_, (**f**) DI × ZnO NP_(100)_. Scale bar = 350 μm. (lb = leaf blade, vb = vascular bundle).

Data presented in Table 9 and Table 10 and Figure 3 and Figure 4 exhibited that leaf blade thickness, length and thickness of midvein, length and width of the vascular bundle were decreased under DI × ZnO NP_(0)_. However, exogenous application of 100 ppm ZnO NP mitigated the detrimental impacts of DI stress on eggplant, in the view of recorded higher or similar values of leaf anatomical traits when compared with FI × ZnO NP_(0)_. The greatest stem anatomy characteristics like length and width of stem, length and width of vascular cylinder, dimensions of the pith (length and width), cortex thickness, and vascular cylinder thickness were recorded with FI × ZnO NP_(100)_, while the lowest values corresponded with DI × ZnO NP_(0)_ (Table 10 and Figure 4). However, ZnO NP (100 ppm) foliar application improved anatomical characteristics of DI plant’s stem when compared with FI × ZnO NP_(0)_.

## 4. Discussion

Drought is among the main constraints in irrigated agriculture, seriously affecting crop production, thus threatening food security [7,48]. These threats to the sustainability of food production are exacerbated by increasing drought episodes in arid regions, including Egypt, particularly when synchronized with salt-alkaline soil [49,50,51]. The combination of salinity-drought stress could limit plant growth via affecting several physio-biochemical processes and causing nutrients deficiencies. Therefore, these stressors could produce significant yield losses. Engineering of adjuvants in the formulation of nanoparticles such as ZnO NP are being deemed as fertilizers, as well as may confer drought stress tolerance [32,34]. 

The current study demonstrated that reducing irrigation up to 60% of ETc resulted in exposure of eggplant to continuous severe water-deficit. This water stress induced losses of membrane integrity and tissue water deficiency, as well as disturbed the photosynthetic capacity of PSII and reduced the relative chlorophyll content (Table 6), which in turn produced substantial impairment of growth-related traits i.e., shoot length, number of leaves plant^−1^, stem diameter, shoot fresh and dry weights, and total plant leaf area (Table 5). A primary response to soil water-deficits is stomatal closure directed via root-to-shoot signals (mainly ABA), directly impacting CO_2_ diffusion into leaf tissues that reduce photosynthesis and diminish eggplant growth [52,53,54]. Stressors like drought and salinity severely inhibit plant growth and productivity via inducing downregulation of the activity of cyclin-dependent kinase enzymes resulting in fewer meristematic cells and cell division and expansion [55,56,57,58,59], in turn reduced numbers of leaves and leaf area (Table 5), coinciding with reduction of relative chlorophyll index and the photosynthetic efficiency of PSII (Table 6). Our results demonstrated that exogenously applied ZnO NP improved growth-related parameters of drought-stressed eggplants. This growth-promotion in ZnO NP-treated plants is most likely related to ZnO NP effects on hormonal signals that modulate root architecture for improved plant adaptation to soil water-deficits [32]. Indeed, ZnO NP enhance gene activity/expression related hormones like ABA and cytokinins, concomitant with regulation of root growth that helped tolerate the drought stress [60]. Moreover, foliar spraying ZnO NP may enhance the restoration of the photosynthetic efficiency, therefore may provide more metabolites/photosynthesis for eggplant growth.

Reduction of available water in the soil and pilling up salts in the soil triggers the accumulation of abscisic acid (ABA) in plant tissues, inducing osmotic stress [16,61,62], and mediated loss of cell turgor due to the lower water availability for cell expansion, thus decreasing the RWC. Furthermore, the reduction of MSI observed in DI-stressed eggplant, may be attributed to the oxidative stress stimulated by excess production of reactive oxygen species (ROS) in plant organelles [50,63,64], inducing lipid peroxidation resulting in a reduction of membrane integrity and loss of cell turgor, indicating that the DI-stressed eggplant leaves experienced cell membrane disintegration [65,66,67]. However, drought-induced damage to cell membrane and reduction of tissue water content was relieved by exogenous-ZnO NP (Table 5), that agrees with those observed by [68] in maize. Eggplant tissue water status promoted in water-stressed plants by foliar spraying ZnO NP proved that ZnO NP may play a role in maintaining cell membrane integrity and increasing RWC as metabolically available water, which could reflect the metabolic processes in plants [69].

Besides decreases of leaf chlorophyll index (SPAD), severe water stress decreased photosynthetic efficiency like the maximum quantum yield of PSII (*F_v_*/*F_m_*) and the electron flow rate through PSII (PI; Table 6). Chlorophyll concentration is related to the photosynthetic efficiency [70,71,72], therefore, the reduction observed in photosynthetic efficiency was associated with chlorophyll degradation [12,73], as occurred in the present study (Table 6). Water-deficits induce degradation of D1 protein that translates into a lower value of *F_v_*/*F_m_* that represents photoinhibition [74], indicating that the damage occurred in the light-harvesting complex of PSII in drought-stressed eggplant. According to our results, exogenous ZnO NP improved eggplant drought stress tolerance, showing that ZnO NP increased SPAD value concurrently with the increase of the chlorophyll fluorescence apparatus (Table 5). These positive findings by foliar-applied ZnO NP may be attributed to stabilizing membrane integrity and increasing the RWC (Table 6) as well as increasing macro- and micronutrients uptake (Table 8) [75] and impeded the chlorophyllase enzyme activity [76] for maintaining the chlorophyll [77], thence increase the photosynthetic efficiency. Along with this line, the authors of [34] reported that application of 100 mg L^−1^ ZnO NP promoted stabilization of the ultrastructure of chloroplast and mitochondria of water-stressed maize which helped to increase the photosynthetic efficiency. This might be linked to the accumulation of osmolytes like proline and sugar content for osmotic adjustment function.

In the present study, drought stress decreased fruit yield and its component (Table 7), which agrees with the results obtained for eggplant [15], tomato [51], and cucumber [78]. Accordingly, they found a reduction in the average weight of fruit, total number of plant fruits, and the total fruit yield when the plant was exposed to water stress. Under drought, exogenous ZnO NP provokes improvements of eggplant water status, the efficiency of PSII, and eggplant growth and biomass that notably reflected on increases in fruit yield and fruit characteristics (number, weight, and length) (Table 7). The favorable results obtained displayed that 100 ppm of ZnO NP (as recommended concentration) was more effective, in the sense that the greatest yield was recorded when applied to fully or deficit irrigated plants. Taken together, these results confirm the previous reports [79] finding that plants require micronutrients in addition to macronutrients for higher development and yield potential. Along with these lines, the authors of [25] demonstrated that sorghum grain yield grown under water stress could be increased up to 183% by ZnO NP. Under limited water conditions, the main goal is providing considerable water-saving and increasing WP [3], which increased by 9.9% by DI in the present study, nevertheless, this improvement in WP is up to 66% by spraying DI-stressed eggplant with ZnO NP (Table 7). In [27] similar findings were reported, indicating that seed and biomass-based WP increased as a result of the supplementation of sunflower plants grown under 50% of irrigation restriction with ZnO NP. Supplemental ZnO NP-induced changes in plant root morphology, increased formation of lateral roots [60], and root biomass [25] presumably increasing water uptake.

Our results exhibited that plants grown under water restriction had a lower concentration of N, K, Zn, and Mn, except for P (Table 8). Soil moisture deficit reduces the nutrients diffusion around the roots as well as nutrients uptake due to decreases in active transport, transpiration flux, and membrane permeability [80]. In sorghum plants, application of ZnO NP either to soil or foliar pathways increased nutrients (N, P, K, Zn, and Mn) concentration, as occurred in the present study (Table 7) [81]. However, leaf concentration of the macro- and micronutrients was modulated by foliar-application of ZnO NP (particularly for 100 ppm, Table 8). Therefore, our results indicated that drought stress has deleterious effects on eggplant nutritional contents, however, exogenous ZnO NP is involved in alleviating these negative impacts. Previous investigations have depicted that ZnO NP could increase macro- and micronutrients in pinto beans [82] and sorghum [25]. In addition to the improvements in membrane stability and plant water status, the improved eggplant nutritional status by exogenous ZnO NP makes eggplant able to attenuate the impacts of DI stress on its growth and productivity.

Plant processes involving genetic, physiological, biochemical, and anatomical mechanisms are responsible for crop performance, these functions are related to the plant’s internal anatomy structures [83]. These enhanced attributes (i.e., growth, yield, SPAD value, photosynthetic capacity, water relations, and WP and nutrients content) in response to externally applied ZnO NP might be linked with the improved leaf and stem anatomical features (Table 9 and Table 10; Figure 3 and Figure 4). ZnO NP foliar application increased leaf and stem anatomical parameters of eggplants grown under deficit irrigation, probably due to improved RWC, cell membrane stability, and nutrients status [75,84].

## 5. Conclusions

According to the findings obtained in the current study, zinc oxide nanoparticles (ZnO NP) foliar application could be a promising practice for improving drought stress tolerance in eggplant cultivated in salt-alkaline soil. These positive effects mainly come from the improved acquisition of macro- and micronutrients, increasing relative water content (RWC), alleviating cell membrane damage (MSI), as well as increasing leaf and stem anatomical traits of eggplants. Eggplant supplemented with ZnO NP elevated SPAD value and chlorophyll fluorescence apparatus (*Fv*/*Fm* and PI). All these factors collectively contributed to better growth and productivity of eggplant grown under deficit irrigation stress. It is important to note that the use of ZnO NP as a foliar spray for eggplant increased water productivity. Thus, our findings gave the utility of using ZnO NP (100 ppm) for ameliorating water stress effects on eggplant production in dry-land agriculture.

## Figures and Tables

**Table 1:** Weather data at Fayoum area, Egypt during the growing season.

Month	Main Temperatures (°C)		RHavg %	U_2_ ms^−1^	Ep mmd^−1^
	Day	Night			
April	33.36	15.92	30.00	1.93	5.60
May	36.50	19.52	31.00	1.90	6.90
June	39.40	20.30	36.00	1.50	7.60
July	40.36	25.90	36.00	2.10	6.90
August	40.40	26.30	37.00	1.80	6.80

RH_avg_ is average relative humidity, U_2_ is average wind speed, and E_P_ is average of measured pan evaporation class A.

**Table 2:** Some initial physical properties of the experimental soil.

Layer (cm)	Particle Size Distribution				Bulk Density (g cm^−3^)	K_sat_ cm h^−1^	FC (%)	WP (%)	AW (%)
	Sand %	Silt %	Clay %	Texture Class					
0–25	73.2	14. 0	12.8	LS	1.52	1.89	27.33	11.73	15.60
25–50	71.2	13.1	15.7	LS	1.47	1.55	26.19	11.13	15.06

FC = field capacity, WP = wilting point, AW = available water, LS = loamy sand, and K_sat_ = hydraulic conductivity.

**Table 3:** Some initial chemical properties of the experimental soil.

**Properties**	**Value**
pH (at a soil: water (*w*/*v*) ratio of 1:2.5)	7.72
ECe (dS·m^−1^; soil—paste extract)	7.33
CEC (cmol_e_ kg^−1^)	11.15
CaCO_3_ (%) Organic matter (%) ESP (exchangeable sodium percentage)	4.85
	1.12
	10.62
**Available Nutrients:**	
N (%)	0.03
P (mg kg^−1^ soil)	511.8
K (mg kg^−1^ soil)	65.9
Fe (mg kg^−1^ soil)	3.54
Mn (mg kg^−1^ soil)	9.6
Zn (mg kg^−1^ soil)	0.60
Cu (mg kg^−1^ soil)	0.51

**Table 4:** Chemical composition of irrigation water.

Ionic Concentration (Meq L^−1^)								EC ^a^ (dS m^−1^)	pH	SAR ^b^
CO_3_^2−^	HCO_3_^−^	Cl^−^	SO_4_^2−^	Ca^2+^	Mg^2+^	Na^+^	K^+^			
0.00	2.8	10.8	5.4	5.0	4.3	8.4	1.3	1.88	7.37	3.99

^a^ EC means the average electrical conductivity, ^b^ SAR means sodium adsorption ratio.

**Table 5:** Effects of foliar application with ZnO nanoparticles (ZnO NP) on vegetative growth of eggplant grown under full (FI) and deficit (DI) irrigation.

Treatments	Shoot Length (cm)	Number of Leaves	Stem Diameter (cm)	Shoot FW Plant^−1^ (g)	Shoot DW Plant^−1^ (g)	Leaf Area Plant^−1^ (dm^2^)
Irrigation (I)	*	*	ns	*	*	*
FI	88.1 ± 1.9 a	61.6 ± 2.5 a	1.3 ± 0.03 a	283.9 ± 15.6 a	55.4 ± 3.3 a	55.5 ± 2.8 a
DI	73.7 ± 2.7 b	47.0 ± 3.3 b	1.2 ± 0.06 b	210.7 ± 10.9 b	42.0 ± 3.4 b	40.3 ± 2.6 b
ZnO NP_(ppm)_	*	*	*	*	*	*
ZnO NP_(0)_	70.8 ± 3.3 c	45.2 ± 3.4 b	1.1 ± 0.07 c	185.2 ± 7.4 c	37.0 ± 3.6 c	34.1 ± 1.8 c
ZnO NP_(50)_	81.0 ± 2.3 b	55.0 ± 4.3 a	1.2 ± 0.04 b	244.6 ± 13.6 b	47.6 ± 3.2 b	50.8 ± 3.4 b
ZnO NP_(100)_	90.8 ± 2.4 a	62.7 ± 3.3 a	1.4 ± 0.04 a	312.0 ± 15.3 a	61.4 ± 3.7 a	58.9 ± 2.5 a
I × ZnO NP	**	**	**	**	**	**
FI × ZnO NP_(0)_	80.8 ± 2.2 bc	52.5 ± 1.3 bc	1.3 ± 0.11 ab	206.0 ± 5.1 d	46.1 ± 3.9 bc	39.8 ± 0.9 c
FI × ZnO NP_(50)_	85.8 ± 1.2 b	67.0 ± 4.4 a	1.3 ± 0.10 ab	286.5 ± 10.7 b	55.2 ± 4.7 ab	61.4 ± 2.4 a
FI × ZnO NP_(100)_	97.5 ± 1.5 a	65.2 ± 4.2 a	1.4 ± 0.04 a	359.2 ± 11.3 a	64.9 ± 5.9 a	65.3 ± 0.4 a
DI × ZnO NP_(0)_	60.7 ± 1.7 d	37.8 ± 5.2 d	1.0 ± 0.08 c	164.3 ± 6.4 e	28.0 ± 2.9 d	28.3 ± 0.4 d
DI × ZnO NP_(50)_	76.2 ± 3.4 bc	43.0 ± 1.9 cd	1.1 ± 0.06 bc	202.8 ± 1.3 d	40.1 ± 0.3 c	40.3 ± 0.5 c
DI × ZnO NP_(100)_	84.2 ± 2.3 b	60.2 ± 5.4 ab	1.4 ± 0.07 a	264.8 ± 11.6 c	57.9 ± 4.6 ab	52.4 ± 3.2 b

** and * indicate, respectively, differences at *p* ≤ 0.05 and *p* ≤ 0.01probability level, and “ns” indicates not significant difference. Means followed by the same letter in each column are not significantly different according to Duncan’s multiple range test (*p* ≤ 0.05).

**Table 6:** Effects of foliar application with ZnO nanoparticles (ZnO NP) on photosynthetic efficiency (*F_v_*/*F_m_*, and PI), relative chlorophyll content (SPAD value), relative water content (RWC), and membrane stability index (MSI) of eggplant grown under full (FI) and deficit (DI) irrigation.

Treatments	*F_v_*/*F_m_*	PI	SPAD Value	RWC%	MSI%
Irrigation (I)	ns	*	*	*	*
FI	0.84 ± 0.00 a	10.8 ± 0.29 a	60.9 ± 0.38 a	78.8 ± 0.99 a	77.5 ± 0.29 a
DI	0.84 ± 0.00 a	8.4 ± 0.38 b	57.3 ± 0.99 b	73.5 ± 1.37 b	74.7 ± 0.83 b
ZnO NP_(ppm)_	*	*	*	*	*
ZnO NP_(0)_	0.83 ± 0.00 b	8.2 ± 0.51 c	56.4 ± 1.3 c	70.8 ± 1.50 c	73.7 ± 0.90 b
ZnO NP_(50)_	0.84 ± 0.00 a	9.7 ± 0.37 b	61.6 ± 0.61 a	81.1 ± 1.30 a	76.6 ± 0.66 a
ZnO NP_(100)_	0.85 ± 0.00 a	10.9 ± 0.43 a	59.3 ± 0.40 b	76.5 ± 0.29 b	77.9 ± 0.48 a
I × ZnO NP	**	**	*	*	*
FI × ZnO NP_(0)_	0.84 ± 0.00 ab	9.8 ± 0.02 bc	60.8 ± 0.12 ab	75.7 ± 0.09 c	76.6 ± 0.31 ab
FI × ZnO NP_(50)_	0.84 ± 0.00 a	10.6 ± 0.62 b	62.4 ± 0.40 a	83.8 ± 0.72 a	78.6 ± 0.51 a
FI × ZnO NP_(100)_	0.85 ± 0.00 a	12.0 ± 0.28 a	59.5 ± 0.63 b	77.0 ± 1.70 bc	77.3 ± 0.31 ab
DI × ZnO NP_(0)_	0.83 ± 0.00 b	6.5 ± 0.24 d	52.1 ± 0.57 c	66.0 ± 1.09 d	70.9 ± 0.44 c
DI × ZnO NP_(50)_	0.83 ± 0.01 a	8.8 ± 0.02 c	60.7 ± 0.57 ab	78.5 ± 1.75 b	77.3 ± 1.25 ab
DI × ZnO NP_(100)_	0.83 ± 0.00 a	9.8 ± 0.45 bc	59.2 ± 1.23 b	76.0 ± 0.64 bc	75.9 ± 0.85 b

** and * indicate, respectively, differences at *p* ≤ 0.05 and *p* ≤ 0.01probability level, and “ns” indicates not significant difference. Means followed by the same letter in each column are not significantly different according to Duncan’s multiple range test (*p* ≤ 0.05).

**Table 7:** Effects of foliar application with ZnO nanoparticles (ZnO NP) on yield, and water productivity (WP) of eggplant grown under full (FI) and deficit (DI) irrigation.

Treatments	Fruit Length (cm)	No. of Fruits Plant^−1^	Fruit Weight (g)	Fruit Yield (t ha^−1^)	WP (kg m^−3^)
Irrigation (I)	*	*	*	*	*
FI	14.2 ± 0.37 a	17.4 ± 0.93 a	67.7 ± 2.87 a	51.3 ± 2.11 a	7.1 ± 0.29 b
DI	12.9 ± 0.39 b	16.0 ± 0.63 b	55.3 ± 1.27 b	42.6 ± 3.03 b	7.8 ± 0.58 b
ZnO NP_(ppm)_	*	*	*	*	*
ZnO NP_(0)_	12.8 ± 0.59 b	13.5 ± 0.66 c	54.7 ± 2.16 c	34.7 ± 2.67 c	5.3 ± 0.25 c
ZnO NP_(50)_	14.1 ± 0.47 a	17.4 ± 0.63 b	69.0 ± 4.29 a	49.4 ± 1.84 b	7.9 ± 0.43 b
ZnO NP_(100)_	13.7 ± 0.38 ab	19.3 ± 0.81 a	60.8 ± 0.95 b	56.7 ± 1.85 a	9.0 ± 0.30 a
I × ZnO NP	**	**	*	*	*
FI × ZnO NP_(0)_	14.5 ± 0.56 ab	13.3 ± 0.95 c	58.7 ± 3.23 b	43.3 ± 0.99 c	5.9 ± 0.14 c
FI × ZnO NP_(50)_	15.2 ± 0.54 a	18.2 ± 0.75 b	82.1 ± 3.15 a	50.1 ± 2.31 b	6.9 ± 0.32 c
FI × ZnO NP_(100)_	12.8 ± 0.48 d	20.8 ± 1.25 a	62.5 ± 0.93 b	60.3 ± 3.09 a	8.3 ± 0.43 b
DI × ZnO NP_(0)_	11.2 ± 0.31 e	13.7 ± 0.99 c	50.7 ± 1.91 c	26.1 ± 0.88 d	4.6 ± 0.28 d
DI × ZnO NP_(50)_	13.0 ± 0.45 bd	16.7 ± 0.99 b	56.0 ± 1.83 bc	48.6 ± 3.04 bc	8.9 ± 0.56 ab
DI × ZnO NP_(100)_	14.5 ± 0.34 abc	17.7 ± 0.56 b	59.2 ± 1.43 b	53.1 ± 0.53 b	9.8 ± 0.10 a

** and * indicate, respectively, differences at *p* ≤ 0.05 and *p* ≤ 0.01 probability level, and “ns” indicates not significant difference. Means followed by the same letter in each column are not significantly different according to Duncan’s multiple range test (*p* ≤ 0.05).

**Table 8:** Concentrations (mg g^−1^ DW) of N, P, K, Zn, Mn, and Fe in eggplant leaves as influenced by foliar application with ZnO nanoparticles (ZnO NP) under full (FI) and deficit (DI) irrigation.

Treatments	N (mg g^−1^ DW)	P (mg g^−1^ DW)	K (mg g^−1^ DW)	Zn (mg g^−1^ DW)	Mn (mg g^−1^ DW)	Fe (mg g^−1^ DW)
Irrigation (I)	*	ns	*	*	*	*
FI	26.30 ± 1.6 a	1.31 ± 0.10 a	28.3 ± 0.81 a	0.32 ± 0.03 a	0.30 ± 0.01 a	0.69 ± 0.03 a
DI	22.30 ± 1.6 b	1.36 ± 0.14 a	23.7 ± 1.16 b	0.27 ± 0.03 b	0.25 ± 0.01 b	0.57 ± 0.02 b
ZnO NP_(ppm)_	*	*	*	*	*	*
ZnO NP_(0)_	18.90 ± 1.3 b	0.90 ± 0.01 c	22.6 ± 1.30 c	0.18 ± 0.01 c	0.25 ± 0.01 c	0.56 ± 0.02 c
ZnO NP_(50)_	26.20 ± 1.6 a	1.42 ± 0.04 b	26.6 ± 1.50 b	0.35 ± 0.01 b	0.29 ± 0.01 b	0.68 ± 0.05 a
ZnO NP_(100)_	27.80 ± 1.2 a	1.68 ± 0.07 a	28.8 ± 0.46 a	0.36 ± 0.01 a	0.30 ± 0.01 a	0.66 ± 0.00 b
I × ZnO NP	**	**	*	**	*	*
FI × ZnO NP_(0)_	21.30 ± 1.6 bc	0.93 ± 0.02 c	25.4 ± 0.67 c	0.20 ± 0.00 d	0.26 ± 0.00 c	0.61 ± 0.01 c
FI × ZnO NP_(50)_	28.10 ± 2.6 a	1.43 ± 0.02 b	29.7 ± 1.04a	0.38 ± 0.00 b	0.32 ± 0.00 b	0.80 ± 0.00 a
FI × ZnO NP_(100)_	29.60 ± 1.0 a	1.57 ± 0.02 b	29.8 ± 1.20 a	0.39 ± 0.00 a	0.33 ± 0.00 a	0.67 ± 0.00 b
DI × ZnO NP_(0)_	16.60 ± 0.9 c	0.89 ± 0.01 c	19.8 ± 0.31 e	0.15 ± 0.00 e	0.23 ± 0.01 e	0.51 ± 0.02 e
DI × ZnO NP_(50)_	24.40 ± 1.6 ab	1.42 ± 0.08 b	23.6 ± 0.21 d	0.33 ± 0.01 c	0.26 ± 0.01 d	0.56 ± 0.00 d
DI × ZnO NP_(100)_	25.90 ± 1.6 ab	1.78 ± 0.12 a	27.8 ± 0.31 b	0.33 ± 0.00 c	0.27 ± 0.00 c	0.65 ± 0.00 b

** and * indicate, respectively, differences at *p* ≤ 0.05 and *p* ≤ 0.01 probability level, and “ns” indicates not significant difference. Means followed by the same letter in each column are not significantly different according to Duncan’s multiple range test (*p* ≤ 0.05).

**Table 9:** Effects of foliar application with ZnO nanoparticles (ZnO NP) on leaf anatomy of eggplant grown under full (FI) and deficit (DI) irrigation.

Treatments	Leaf Blade Thickness (μm)	Midvein Length (μm)	Midvein Thickness (μm)	Vascular Bundle Length (μm)	Vascular Bundle Width (μm)
I × ZnO NP	*	*	*	*	*
FI × ZnO NP_(0)_	200	3625	3000	2050	750
FI × ZnO NP_(50)_	175	3375	3000	1875	625
FI × ZnO NP_(100)_	200	4000	3750	2400	875
DI × ZnO NP_(0)_	175	2875	2500	1500	625
DI × ZnO NP_(50)_	225	3250	3175	1750	750
DI × ZnO NP_(100)_	250	3500	3375	1875	750

* indicate respectively differences at *p* ≤ 0.05 probability level, and “ns” indicates not significant difference. Means followed by the same letter in each column are not significantly different according to Duncan’s multiple range test (*p* ≤ 0.05).

**Table 10:** Effects of foliar application with ZnO nanoparticles (ZnO NP) on stem anatomy of eggplant grown under full (FI) and deficit (DI) irrigation.

Treatments	Dimensions of Stem (μm)		Dimensions of Vascular Cylinder (µm)		Dimensions of Pith (µm)		Cortex Thickness (µm)	Vascular Cylinder Thickness (µm)
	Length	Width	Length	Width	Length	Width		
I × ZnO NP	*	*	*	*	*	*	*	*
FI × ZnO NP_(0)_	5000	4875	3625	3375	2375	1750	625	625
FI × ZnO NP_(50)_	5450	4750	4050	3325	3125	2350	700	500
FI × ZnO NP_(100)_	6250	6125	4500	4250	3500	2750	925	550
DI × ZnO NP_(0)_	3825	3750	2875	2450	2400	1750	575	375
DI × ZnO NP_(50)_	4625	4375	3250	2875	2375	2250	750	400
DI × ZnO NP_(100)_	6050	5200	4325	4000	3375	3125	900	500

* indicate respectively differences at *p* ≤ 0.05 probability level, and “ns” indicates not significant difference. Means followed by the same letter in each column are not significantly different according to Duncan’s multiple range test (*p* ≤ 0.05).

## Data Availability

The data presented in this study are available upon request from the corresponding author.
